# Radiomic Analysis in Pituitary Tumors: Current Knowledge and Future Perspectives

**DOI:** 10.3390/jcm13020336

**Published:** 2024-01-07

**Authors:** Fabio Bioletto, Nunzia Prencipe, Alessandro Maria Berton, Luigi Simone Aversa, Daniela Cuboni, Emanuele Varaldo, Valentina Gasco, Ezio Ghigo, Silvia Grottoli

**Affiliations:** Division of Endocrinology, Diabetes and Metabolism, Department of Medical Sciences, University of Turin, 10126 Turin, Italy; fabio.bioletto@unito.it (F.B.); nunzia.prencipe@unito.it (N.P.); alessandromaria.berton@unito.it (A.M.B.); luigisimone.aversa@unito.it (L.S.A.); daniela.cuboni@unito.it (D.C.); emanuele.varaldo@unito.it (E.V.); valentina.gasco@unito.it (V.G.); ezio.ghigo@unito.it (E.G.)

**Keywords:** PitNET, pituitary tumor, pituitary adenoma, radiomics, consistency, invasiveness, histology, recurrence, treatment response

## Abstract

Radiomic analysis has emerged as a valuable tool for extracting quantitative features from medical imaging data, providing in-depth insights into various contexts and diseases. By employing methods derived from advanced computational techniques, radiomics quantifies textural information through the evaluation of the spatial distribution of signal intensities and inter-voxel relationships. In recent years, these techniques have gained considerable attention also in the field of pituitary tumors, with promising results. Indeed, the extraction of radiomic features from pituitary magnetic resonance imaging (MRI) images has been shown to provide useful information on various relevant aspects of these diseases. Some of the key topics that have been explored in the existing literature include the association of radiomic parameters with histopathological and clinical data and their correlation with tumor invasiveness and aggressive behavior. Their prognostic value has also been evaluated, assessing their role in the prediction of post-surgical recurrence, response to medical treatments, and long-term outcomes. This review provides a comprehensive overview of the current knowledge and application of radiomics in pituitary tumors. It also examines the current limitations and future directions of radiomic analysis, highlighting the major challenges that need to be addressed before a consistent integration of these techniques into routine clinical practice.

## 1. Introduction

Pituitary neuroendocrine tumors (PitNETs), also commonly referred to as pituitary adenomas, represent a relatively common pituitary disease, accounting for approximately 15% of all intracranial neoplasms [1]. Most of these tumors exhibit a benign behavior, but may be characterized by more aggressive local growth in a certain percentage of cases [1,2,3]. In addition to local mass effects, they can exhibit a wide range of systemic manifestations due to their impact on the endocrine system, either due to hypersecretory syndromes or to hypopituitarism [1].

The characterization and the management of pituitary tumors require a comprehensive understanding of their clinical, biochemical, and radiological features, which are crucial for accurate diagnostic evaluation and treatment planning [1]. With regard to radiological assessment, magnetic resonance imaging (MRI) represents the imaging technique of choice to evaluate tumor size and invasiveness [1]; moreover, the characteristics of MRI signals can provide, in some circumstances, further insights into specific tumor characteristics, which can be predicted by the qualitative assessment of signal intensity on T1-weighted images (T1WIs), T2-weighted images (T2WIs), and/or contrast-enhanced T1-weighted images (CE-T1WIs) [1,4,5,6,7].

In recent years, radiomics has emerged as a promising tool in medical imaging, offering novel insights into tumor characterization and prognostication [8]. Radiomic techniques involve the extraction of a large number of quantitative imaging features from medical images, followed by the application of advanced statistical and machine-learning techniques to derive different predictive models [8,9]. By harnessing the wealth of information contained within medical images, radiomics enables a non-invasive and quantitative analysis of tumor characteristics that may be otherwise imperceptible to the human eye [8,9].

**Figure 1:**
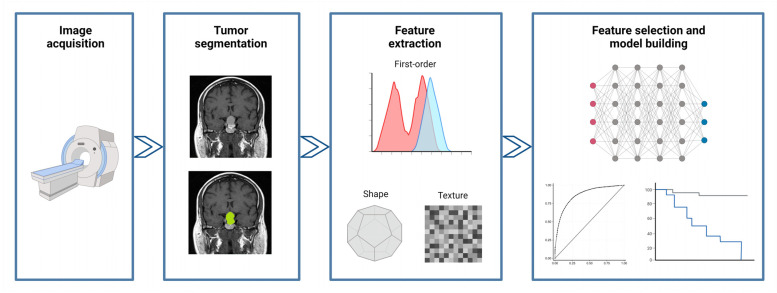
General description of the workflow that characterizes a radiomic study. Created with BioRender.com.

The general workflow of a radiomic study consists of several steps (Figure 1). After image acquisition, it is first necessary to define the region of interest (ROI) corresponding to the target lesion; afterwards, several types of quantitative features (such as first-order statistics, shape-based features, and texture features) can be extracted from the segmented ROI using dedicated software; finally, specific statistical tools—often based on a machine-learning approach—are used to select the most relevant features and to build a predictive/prognostic model.

In the context of pituitary tumors, radiomic analysis holds promising potential to enhance diagnostic accuracy, predict treatment response, and facilitate personalized medicine. The integration of radiomics with clinical, histopathological, and genomic data has the potential to unlock a deeper understanding of pituitary tumor biology and improve patient outcomes. This review paper aims to provide an overview of the existing literature on radiomic analysis in PitNETs. We discuss how radiomic features have shown promise in predicting pituitary tumors’ macroscopic and microscopic characteristics. Additionally, we delve into the predictive value of radiomics in the assessment of treatment response and recurrence risk.

## 2. Prediction of Tumor Consistency

PitNETs can be broadly classified as soft or fibrous, depending on their hardness at a macroscopic level. Fibrous PitNETs are relatively less common than softer ones, with a prevalence that has been estimated as approximately 18% (range of 6–73%) [10]. The fibrous consistency of a pituitary tumor is influenced by several factors, including higher collagen content and poor cellularity [7,11]; furthermore, previous surgical and non-surgical interventions could induce fibrous alterations and tissue deformations [7,11]. Tumor consistency is an important parameter to consider during surgical planning; soft adenomas, in fact, can be properly removed through aspiration and curettage, while those with a hard and fibrous consistency are less prone to exeresis [5]. Hence, the distinction between soft and hard PitNETs before surgery is important to facilitate surgical preparation and prevent the need for multiple surgical interventions.

Previous studies found a correlation between the consistency of tumors and their signal intensity on T2WIs. In fact, a hypointense signal intensity on T2WIs seems to correlate with hard PAs, probably due to their higher percentage of collagen content [5,6,7], while soft adenomas appear hyperintense on the same sequence, likely because of the higher content of water and/or cystic components [5,6,7]. In recent years, radiomic features have also been evaluated by some authors as potential predictors of tumor consistency.

In 2019, Zeynalova and colleagues [6] performed a study in which they evaluated the accuracy of an artificial neural network in predicting tumor consistency in 55 patients with PitNETs (42 soft and 13 fibrous macroadenomas, either functioning or non-functioning) and compared its performance to the one of the signal intensity ratio on T2WIs. The reference standard for tumor consistency was a combination of retrospective judgment provided by two neurosurgeons, together with the histopathological assessment of the collagen content and density performed by three different pathologists. In this analysis, the artificial neural network correctly classified 72.5% of tumors regarding consistency; the area under the receiver operating characteristic curve (AUC) was equal to 0.71 and was significantly superior to that of the signal intensity ratio (AUC 0.55, *p* = 0.02). These results were internally validated by 10-fold cross validation. Some of the main limitations of this research include the lack of a separate validation set, the manual segmentation of planar images, and finally the need to obtain samples from multiple consecutive sections of the same fibrous adenomas, due to the limited number of this type of adenomas.

In a similar way, a year later, Cuocolo et al. [12] performed a study on 89 patients operated for PitNETs (68 soft and 21 fibrous macroadenomas, either functioning or non-functioning), in order to evaluate if radiomic features were able to accurately predict tumor consistency. The reference standard adopted for the definition of consistency was the opinion of two independent neurosurgeons. A total of 1118 radiomic texture features were extracted from T2WIs, and 14 of them were finally retained as the most relevant ones based on feature stability, variance, intercorrelation, and recursive feature elimination. An ensemble learning meta-algorithm (the Extra Trees Classifier) was finally applied to build a predictive model, which showed an AUC of 0.99. The sensitivity, specificity, and accuracy of the model were 100%, 87%, and 93%, respectively.

Regardless of T2WIs, other data collected in this field have suggested that heterogeneous patterns on T1WIs or CE-T1WIs may also relate to soft PitNETs, possibly reflecting high vascularization and microhemorrhages, while fibrous tumors would more often demonstrate a homogeneous enhancement [13]. Therefore, some subsequent studies have attempted to apply radiomic analysis simultaneously to a broader range of MRI sequences, including T1WIs, CE-T1WIs, and T2WIs. Among these, a study published in 2022 by Wan et al. [11] analyzed radiomic features from the volume of interest on both individual T1WI/CE-T1WI/T2WI sequences and their combinations. The images were delineated via an automated 3D segmentation method. For this research, 156 patients with PitNETs were enrolled (104 soft and 52 hard macroadenomas), who were randomly divided into a training (70%) and a test (30%) cohort. The standard reference was exclusively the opinion of the neurosurgeon and his assistant, collected immediately after surgery. The authors noted that shape features appeared less useful than texture and wavelet features, thus confirming that multiparametric MRI could provide more complete information to evaluate the tumor consistency of PitNETs. In detail, the analysis of integrated features based on histograms, textures, and wavelets in the 3D domain demonstrated that hard PitNETs (generally fibrous and with greater structural variations within) had greater heterogeneity in their texture compared to soft PitNETs. Overall, the predictive performance of the final radiomics model had an AUC of 0.90; the accuracy, sensitivity, and specificity were 87%, 83%, and 87%, respectively.

Interestingly, the relevance of radiomic features in the prediction of tumor consistency was also assessed specifically in the context of GH-secreting adenomas. In patients with acromegaly, neurosurgical intervention represents the first therapeutic approach in most cases, and tumor consistency represents one of the main factors influencing surgical outcome [14,15]. Therefore, in 2019, Fan and colleagues [16] conducted a study specifically designed to identify a radiomic signature for the prediction of the consistency of GH-secreting tumors. They analyzed 158 naïve acromegalic patients, whose lesions were retrospectively classified into hard or soft PitNETs by two experienced neurosurgeons viewing high-quality surgical videos. The patients were randomized into a training cohort (100 patients) and an internal validation cohort (58 patients); finally, 30 additional patients from other centers were analyzed for external validation. In total, 38% of patients in the primary cohort and 34% in the internal validation cohort harbored hard tumors. A radiomic analysis was performed with an initial extraction of 4683 quantitative features from T1WIs, T2WIs, and CE-T1WIs; an elastic net selection algorithm was adopted, and four features were finally identified as the most relevant ones for the prediction of tumor consistency, with AUC values of 0.84 and 0.76 in the training and internal validation cohort, respectively. This performance was further enhanced when integrating radiomics data with Knosp grade, with AUC values of 0.83 and 0.81 in the training and internal validation set, respectively. This combined classifier was confirmed to perform adequately also in the multicenter external validation cohort, with an AUC of 0.89 and an accuracy of 86.7%.

## 3. Prediction of Tumor Invasiveness

Pre-operative radiological information on the invasiveness of PitNETs is essential for the planning of appropriate surgical strategies and the long-term management of patients. At present, the Knosp grade represents the most used classification to define the parasellar extension of PitNETs [17]. PitNETs with a Knosp grade 4 are always characterized by cavernous sinus (CS) invasion, while those with Knosp grades 0 and 1 are not. However, the pre-operative assessment of CS invasion often remains uncertain in case of Knosp grades 2 and 3 [18].

A first attempt to predict pre-surgical CS invasion by applying radiomic analyses on T2WIs and CE-T1WIs was performed by Niu et al. in 2019 [19]. In this single-center retrospective analysis, a total of 194 patients with Knosp grade 2 and 3 PitNETs were enrolled; two distinct sets of 97 patients were then created and used as a training set and as a test set. A support vector machine algorithm was applied to the training set to derive a radiomics signature; then, a nomogram incorporating both the radiomic signature and other clinical–radiological risk factors was built in the training set and then validated in the test set. After feature selection, the final radiomic signature was composed of three quantitative features extracted from CE-T1WIs, represented by tumor sphericity, the degree of wrapping of the internal carotid artery, and the gray intensity of tumor region. On the other hand, the predictive parameters included in the clinical–radiological model were the Knosp grade, periarterial enhancement, and inferolateral venous compartment obliteration. In terms of performance, the radiomic signature showed AUC values of 0.85 and 0.83 in the training and in the test sets, respectively; the same values were observed also for the clinical–radiological model. Notably, the final nomogram incorporating both the radiomic signature and the clinical–pathological risk factors yielded an AUC of 0.90 in the training set and 0.87 in the test set, performing significantly better than the clinical–radiological model alone.

A few years later, Zhang et al. [20] analyzed the radiomic 3D features of pre-operative CE-T1WIs of 196 patients awaiting neurosurgical intervention for PitNETs in search of predictive parameters for invasive behavior. The reference standard for invasiveness was the assessment of the Knosp grade in the coronal scan by two physicians under double-blind conditions; tumors with a Knosp grade ≥ 3 were defined as invasive. As in the previous study, the patients were divided into a training and validation set, in a 9:1 ratio. In the training set, composed of 176 patients, 95 cases (54%) were defined as invasive; in the validation set, composed of 20 patients, 12 cases (60%) were defined as invasive. A support vector machine model was thus built for predicting tumor invasiveness, and yielded an AUC of 0.86 (with 80% accuracy) and an AUC of 0.73 (with 85% accuracy) in the training and validation sets, respectively.

Interestingly, in recent years, Dynamic Contrast-Enhanced MRI (DCE-MRI) has made it possible to better evaluate the microvascular state of tumor lesions and, in particular, the permeability of microvessels. This imaging modality can be used to detect the distribution of the contrast medium in the blood vessels of the lesions as well as in the extracellular extravascular space, obtaining as a result derived maps, such as the volume transfer constant (Ktrans), the extravascular extracellular volume fraction (Ve), and the rate constant (Kep), thus providing a quantifiable illustration of the vascular heterogeneity of the lesion [21]. Based on this knowledge, Liu et al. [21] applied the principles of radiomic texture analysis to DCE-MRI-derived maps, with the aim of evaluating their performance in the prediction of PitNET aggressiveness. Overall, 50 patients were enrolled, and aggressiveness was defined as the presence of any of the following three criteria: (i) Knosp grade 3–4 and modified Hardy–Wilson classification grade III–IV or stage C–E; (ii) evidence of dural invasion, sellar floor invasion, sphenoid sinus invasion, or cavernous sinus invasion on both sides at intraoperative exploration; and (iii) evidence of dural invasion or sellar floor invasion at histological examination. Radiomic features were extracted from Ktrans, Ve, and Kep maps and, after dimensionality reduction, the best performing ones were combined into an integrated predictive model, which showed a promising performance in the distinction of aggressive and non-aggressive PitNETs, with an AUC of 0.96, a sensitivity of 94.4%, and a specificity of 90.6%.

## 4. Prediction of Hormonal Secretion Patterns

The use of radiomics has been recently explored as a potential predictor of hormonal secretion patterns. Clearly, this type of evaluation has a relatively limited interest in clinical practice, given that the biochemical phenotype of pituitary tumors can be easily assessed directly through laboratory findings. Nevertheless, exploring the correlation between the MRI texture features of the adenoma and its associated secretory pattern could still be a topic of interest for a more in-depth application of radiomic analyses in the context of PitNETs.

In 2022, Baysal et al. [22] conducted a study aimed at detecting hormone secretion profiles in pituitary adenomas based on T2WI radiomic analysis. The study included a retrospective cohort of 130 patients with pituitary adenomas, and the patients were classified according to seven hormone secretion profiles, including non-functioning pituitary adenomas (NFPAs), GH-secreting adenomas, prolactinomas, ACTH-secreting adenomas, FSH/LH-secreting adenomas, TSH-secreting adenomas, and pluri-hormonal secreting adenomas. The authors developed a multivariable diagnostic prediction model using artificial neural networks for each of the seven hormone secretion profiles. The performance of these predictive models was evaluated by the AUC, and a reliable predictive performance was achieved for most hormone profiles. In particular, the observed AUC values were equal to 0.87 for NFPAs, 0.89 for GH-secreting adenomas, 0.95 for prolactinomas, 0.94 for ACTH-secreting adenomas, 0.96 for FSH/LH-secreting adenomas, 0.95 for TSH-secreting adenomas, and 0.74 pluri-hormonal secreting adenomas.

## 5. Prediction of Histopathological Features

In patients with PitNETs, various histopathological features have been demonstrated to harbor a significant diagnostic and prognostic value. The pre-operative knowledge of the presumptive histopathological features of a pituitary tumor can be of help for the planning of appropriate surgical strategies and the long-term management of these patients, and radiomic analyses have been used, in recent years, in an attempt to build predictive models.

In recent years, PitNETs have been classified into three main families according to the expression of specific transcription factors (Tpit, Pit-1, and SF-1). Based on this classification, in 2020, Peng and colleagues [23] aimed to develop a radiomics model to accurately classify these different PitNET subtypes. The study included 235 pathologically diagnosed pituitary tumors; T1WIs, T2Wis, and CE-T1WIs were used for the radiomic analysis, and a total of 788 radiomic features were extracted from each of these sequences. After dimensionality reduction, 18 radiomic features were retained as the most informative ones; three different machine learning models (support vector machine, k-nearest neighbors, and naïve Bayes) were then trained based on the selected variables. The best predictive performance was obtained by the support vector machine classifier on T2WIs; notably, this model achieved a balanced accuracy of 89% and an AUC of 0.95 in the classification of PitNET subtypes. The k-nearest neighbors and naïve Bayes models had a slightly lower performance, with an AUC of 0.93 for both.

In the context of NFPAs, Rui et al. [24] explored the possibility of using radiomic features to differentiate silent corticotroph adenomas (SCAs) from other histological NFPA subtypes. A total of 302 patients operated for NFPAs were enrolled, including 146 patients with SCAs; these patients were then randomly allocated to a training and test set in a 4:1 ratio. The authors examined various clinical and radiomic characteristics, extracted from T1WIs, T2WIs, and CE-T1WIs, and compared them between patients in the SCA and in the non-SCA groups. These characteristics were combined through several machine learning algorithms, the best of which provided an AUC of 0.93 for the distinction between the two NFPA subtypes. Notably, when examining the feature importance of this model, radiomics was the most important one, followed by sex, age, and other less relevant parameters.

In another study on the same topic, Wang et al. [25] confirmed the diagnostic value of radiomic analyses for the pre-operative prediction of SCAs among patients with NFPAs. The study enrolled an initial dataset of 260 patients, consisting of 72 cases with SCAs and 188 cases with other histological NFPA subtypes. An additional 35 patients, comprising 6 SCAs and 29 non-SCAs, were included as an external validation cohort. The SCA group exhibited a higher proportion of female patients and a greater prevalence of multiple microcystic changes, and was characterized by an increased invasiveness, as indicated by the higher Knosp grades. The radiomics model, based on the extraction of features from T1WIs, T2WIs, and CE-T1WIs, demonstrated a high diagnostic performance, with an AUC of 0.93 in the internal dataset and 0.94 in the external dataset. Moreover, a SCA scale was proposed and achieved AUC values of 0.88 and 0.90 in the internal and external datasets, respectively.

Instead of SCAs, Zhang et al. [26] applied radiomic analyses to distinguish between null cell adenomas (NCAs) and other histological NFPA subtypes. They enrolled 112 patients operated for NFPAs; a total of 1482 quantitative imaging features were extracted from pre-operative T1WI and CE-T1WI MRI. A support vector machine algorithm was trained to create a predictive model on a training set, and its performance was then validated on an independent test set. The results showed that T1WI features had AUC values of 0.83 and 0.80 for the training and test sets, respectively, while CE-T1WI features did not provide any significant additional contribution. A nomogram was also created by integrating sex and the T1WI radiomic signature, and demonstrated a good calibration in both the training and test sets, with concordance indices of 0.85 and 0.86, respectively.

Shifting to the field of acromegaly, it is known that the histological evaluation of the granulation pattern is of key importance because of its prognostic and therapeutic implications. In the study by Park et al. [27], the authors evaluated 69 acromegalic patients, 50 with a densely granulated adenoma and 19 with a sparsely granulated adenoma. The study aimed to determine whether radiomic features could predict the granulation pattern of GH-secreting pituitary adenoma patients. A total of 214 radiomic features were extracted from T2WIs, and a radiomics model was constructed using a least absolute shrinkage and selection operator (LASSO) logistic regression model with cross-validation. This radiomics model achieved an AUC of 0.83, with 73.7% accuracy, 74.0% sensitivity, and 73.9% specificity for predicting the granulation pattern. The radiomics model outperformed qualitative T2 signal intensity assessment and T2 relative signal intensity evaluation, with a significantly better performance.

In another study on the same topic, Liu et al. [28] evaluated 49 acromegalic patients, 24 with a densely granulated adenoma and 25 with a sparsely granulated adenoma. After the extraction of radiomic features from T1WIs, T2WIs, and CE-T1WIs, the researchers identified nine texture parameters that showed significant differences between the two groups. In particular, the results showed that the T1WI signature had the highest diagnostic performance, with an AUC of 0.92; the accuracy, sensitivity, and specificity for differentiating densely granulated and sparsely granulated adenomas were 85.7%, 72.0%, and 100.0%, respectively. The CE-T1WI signature also obtained a relatively high efficacy with an AUC of 0.89, and combining the texture features of T1WIs and CE-T1WIs resulted in a radiomic signature with a high predictive performance and an AUC of 0.91.

Radiomic features have also been evaluated as possible predictors of histopathological markers of aggressiveness, such as Ki67 and p53. In a study by Ugga et al. [29], the authors evaluated the possibility of distinguishing, through radiomic analysis, patients with a low (Ki67 < 3%) or a high (Ki67 ≥ 3%) proliferation index. A total of 89 patients who had a surgical resection of a pituitary adenoma (either functioning or non-functioning) were included in this study and were divided into a training group and a test group. A set of 1128 quantitative imaging features was extracted from T2WI sequences. Various supervised feature selection methods were adopted to identify the most informative features and, subsequently, a k-nearest neighbors classifier was used to distinguish between PitNETs with a low or high proliferation index. Overall, the k-nearest neighbors model obtained a good predictive performance in the distinction between classes, correctly classifying 91.7% of patients in the test group, with an AUC of 0.87.

Similarly, Li and colleagues [30] also aimed to explore the potential of using radiomic analysis to predict the Ki67 proliferation index in PitNETs. The threshold for the definition of high tumor proliferation was the same (Ki67 ≥ 3%). A total of 1214 patients from four centers were enrolled; three centers accounted for 1149 of them, who were randomly assigned to a training and testing set in a 7:3 ratio; the fourth center provided 65 patients, who were used as a separate validation cohort. A total 1409 radiomic features were extracted from T1WIs, T2WIs, and CE-T1WIs. Afterwards, the authors selected the most relevant parameters through a backward elimination method followed by a LASSO regression model. A bagging decision tree machine learning classification algorithm was then used to build a final radiomic signature, which combined T1WI and CE-T1WI imaging and showed an AUC of 0.93 in the training set, 0.83 in the testing set, and 0.83 in the independent validation set. Finally, a combined clinical/radiomic nomogram was created, with age and Hardy’s grade being retained as significant predictive factors together with the previously obtained radiomic scores.

Wang et al. [31] also published a study with a similar design, in which the authors aimed to evaluate the clinical value of combining shape and texture radiomic features in assessing the aggressiveness of pituitary adenomas. In this case, the definition of aggressive behavior was not based solely on Ki67, but was defined as the presence of at least two of the following three characteristics: Ki67 ≥ 3%, high mitotic count (≥2/10 high power fields), or positive staining for p53. A total of 246 patients with pituitary adenomas (84 aggressive and 162 non-aggressive) were included. The patients were divided into a training and a test set (193 patients in the former and 53 patients in the latter). Shape-related and textural radiomic features extracted from CE-T1WIs were compared between the aggressive and non-aggressive groups, and logistic regression models were constructed to predict tumor aggressiveness. The results showed that the combination of the four selected features along with the Knosp grade yielded an AUC value of 0.94 in the test set; the sensitivity and the specificity in the identification of aggressive PitNETs were 94.4% and 82.9%, respectively.

The correlation between radiomic markers and PitNET proliferative activity was also assessed by Fan et al. [32]; differently from other reports, however, the authors focused their analysis specifically on patients with acromegaly. Overall, 138 patients were enrolled and randomly assigned into a training and a test set (which comprised 90 patients and 48 patients, respectively). The definition of high proliferative activity was based on a Ki67 index ≥ 3%. A total of 4684 radiomic features were extracted from T1WIs, T2WIs, and CE-T1WIs; subsequently, elastic net and recursive feature elimination algorithms were utilized to select the most relevant ones, and a support vector machine method was used to construct a radiomic signature. The final signature was based on 17 features and demonstrated AUC values of 0.96 and 0.89 for the discrimination between PitNETs with high vs. low proliferative index in the primary cohort and the validation cohort, respectively. As a further step, this signature was integrated with clinical and biochemical patient data to build a nomogram, which showed an AUC value of 0.94 in the primary cohort and 0.91 in the validation cohort.

## 6. Prediction of Response to Surgical Treatment

Surgery is the treatment of choice in most patients with PitNETs, with the exception of prolactinomas, which can be treated with dopamine agonists, and asymptomatic NFPAs (i.e., not associated with any endocrinological/neurological alteration), which can be managed by surveillance alone [1]. The aim of the surgery is to achieve a gross total resection or, if not feasible, a subtotal resection of the tumor. Despite advances in surgical techniques, however, the percentage of patients that recur (after a gross total resection) or progress (after a subtotal resection) is not negligible [1,33], with estimated rates between 0.7% and 3.4% of patients per year [33]. The reported prognostic factors include age, gender, tumor size, tumor invasion (including the involvement of the cavernous sinuses or dura), and tumor histopathology (including the assessment of proliferation markers, such as Ki67 and MIB-1 index) [33]. Moreover, in recent years, the potential of radiomic features to predict tumor recurrence after surgery has been explored by some authors.

Galm et al. [34] conducted a retrospective observational study involving 78 patients operated on for NFPAs. In this study, the authors performed an MRI texture analysis on coronal T1WIs before surgery and discovered that the mean, median, mode, minimum, and maximum pixel intensities were associated with the risk of pituitary tumor recurrence or progression after surgery. Patients whose tumor mean pixel intensity exceeded the population’s median had a hazard ratio of 0.44 for recurrence or progression compared to tumors below the median. This suggests that MRI texture analysis could predict the risk of tumor recurrence or progression in these patients. Conversely, no correlation was found with other clinical, radiological, and histopathological factors.

In a similar study design, Machado et al. [35] analyzed the post-surgical outcomes of 27 NFPA patients; 10 of them experienced tumor recurrence during follow-up, while the other 17 patients did not. All 27 patients were previously treated and monitored by the same multi-professional team at the same healthcare center. The authors conducted an analysis based on pre-operative CE-T1WIs, from which 255 radiomic features were extracted using two extraction modalities (2D and 3D segmentation). Among these, 6 two-dimensional and 13 three-dimensional features were found to be significantly different between the two outcome groups. Five machine-learning algorithms were then trained to predict recurrence; the k-nearest neighbors classifier was the one that achieved the best results using 2D features, with an AUC of 0.98 and an accuracy of 92.6%. On the other hand, the random forest classifier was the one that achieved the best performance using 3D features, with an AUC of 0.96 and an accuracy of 96.3%. Notably, the 3D-feature-based models achieved their best performances using significantly fewer features (between 3 and 5 features) in comparison to the 2D-feature-based models (between 6 and 15 features).

Another study on the same topic was performed by Zhang et al. [36]. In this paper, the authors developed a radiomic model for predicting the progression/recurrence of NFPAs after surgery. A total of 50 eligible patients were enrolled, and pre-operative T2WIs and CE-T1WIs were used for radiomic feature extraction. A support vector machine classifier was adopted for feature selection and model creation; a radiomic score was thus derived, based on the three most relevant radiomic features. The support vector machine classifier showed an AUC of 0.78 and an accuracy of 82% in distinguishing progressive/recurrent NFPAs from non-progressive/recurrent ones; the radiomic score further improved this performance, with an AUC of 0.87. This score was demonstrated to be an independent predictor of progression/recurrence also after accounting for other relevant clinical parameters at multivariable Cox regression analysis.

In a more recent study, comprehensive of all kinds of pituitary adenomas, both functioning and non-functioning, Zhang and colleagues [37] assessed the advantage of including radiomic features in the prediction of post-surgical recurrence compared to the performance of a model based on clinical and histopathological predictors alone. A total of 168 patients were enrolled, divided into a training and a test set in a 7:3 ratio; 1130 radiomic features were extracted from CE-T1WI, and LASSO regression was applied to select the most relevant ones. The prediction of post-surgical outcomes was modeled using two multilayer perceptron classifiers. The first one was based solely on clinicopathological features (age, tumor height, tumor invasion, and tumor residue) and achieved an AUC of 0.75 and 0.74 in the training and in the test set, respectively; the second one integrated to these clinicopathological parameters also four radiomic features identified by LASSO regression, and achieved a slightly better performance with an AUC of 0.80 and 0.78 in the training and in the test set, respectively.

Finally, Fan et al. [38] proposed a radiomics model which specifically focused on the prediction of post-surgical remission in patients with invasive functional adenomas. Overall, 163 patients were enrolled and divided into a training (108 patients) and a test set (55 patients). The definition of post-surgical remission was based on post-operative hormone levels. A total of 13,950 radiomic features were first extracted from T2WIs, T1WIs, and CE-T1WIs; 7 of them were then selected as the best performing ones, all derived from CE-T1WIs. A radiomic signature was built using a support vector machine and showed AUC values of 0.83 and 0.81 in the training cohort and test cohort, respectively; these performances were significantly higher than the model based on clinical features (AUC values of 0.67 and 0.76). The integration of clinical and radiomic parameters did not further improve the predictive power of the radiomic signature alone, with AUC values confirmed at 0.83 and 0.81 in the two sets, respectively.

## 7. Prediction of Response to Non-Surgical Therapies

In recent years, a substantial number of studies have been conducted to identify the predictors of sensitivity or resistance to non-surgical therapies, especially for functioning tumors [4,39,40,41,42,43]. Notably, the majority of these studies have concentrated on predictors of response to somatostatin receptor ligands (SRLs) in GH-secreting tumors, with the goal of customizing treatments [4,39,40]. These predictors include characteristics such as intensity in T2WIs or histological granulation patterns.

Lately, radiomics has also begun to play an important role. In a recent study, Kocak et al. [44] enrolled 47 patients with acromegaly and proposed that the machine-learning-based quantitative texture analysis of T2WIs holds the potential to predict the response to SRLs more effectively than the quantitative and qualitative assessment of relative signal intensity and the evaluation of immunohistochemical granulation patterns. Notably, using the k-nearest neighbors classifier, they were able to correctly classify 85.1% of the patients, with an AUC of 0.85.

A similar study has been conducted by Galm et al. [45], who retrospectively analyzed 64 acromegalic patients and their response to SRLs in terms of IGF-1 normalization. In this case, the extraction of radiomic features was performed on T1WIs. Various texture parameters were extracted and analyzed, and the authors found that subjects with a maximum pixel intensity above the median exhibited increased odds of achieving IGF-1 normalization (odds ratio (OR) of 5.96; 95% confidence interval (95%CI) of 1.33–26.66). This effect persisted even after adjusting for various potential response predictors, such as age, sex, pre-operative tumor diameter, pre-operative IGF-1 levels, and Ki67 index, although it was not influenced by granulation density.

Some data are also available in the context of prolactinomas. Park et al. [46] studied the radiological features of 177 prolactinoma patients, evaluating radiomic features as possible predictors of response to treatment with dopamine agonists (DAs). To this scope, the authors extracted texture data from T2WIs, built an ensemble classifier on a training set, and evaluated its performance on a separate test set. Overall, in the test set, the final model showed an AUC of 0.81; the accuracy was 77.8%, the sensitivity 78.6%, and the specificity 77.3%. On the other hand, their study also suggested that conventional imagining parameters, such as cystic/hemorrhagic changes or T2 relative signal intensity alone, could not reliably predict DA response.

Finally, radiomic analysis has also been assessed as a potential predictor of response to radiotherapy. In a study conducted by Fan et al. [47] in 2019, 57 acromegaly patients who had undergone radiotherapy post-neurosurgery were examined. The researchers constructed various models, based on clinical and radiomic features, to non-invasively predict the response to radiotherapy, defined as the achievement of disease remission at 3 years. The model based only on clinical variables was characterized by an AUC of 0.86; the radiomics model, based on features extracted from T1WIs, T2WIs, and CE-T1WIs, achieved a higher discrimination ability, with an AUC of 0.92. The combination of clinical and radiomic data further improved the predictive performance, with an AUC of 0.96.

## 8. Conclusions

In conclusion, the application of radiomic analysis in the study of PitNETs has started to demonstrate its potential to support the diagnosis, characterization, and management of these diseases. In fact, radiomics offers a non-invasive, quantitative tool that has the potential to unlock hidden insights into tumor characteristics, improving diagnostic accuracy and aiding in treatment decisions. The association between radiomic features and the several key aspects of PitNETs has been explored by various authors. In particular, their role in the prediction of tumor consistency, invasiveness, secretory patterns, histopathological features, and treatment response has been assessed. This review paper provided an overview of the existing literature on radiomic analysis in PitNETs; relevant studies have been discussed and are summarized in Table 1.

As the field of radiomics continues to evolve, its integration with clinical, histopathological, and genomic data promises to enhance our understanding of PitNET biology and improve patient outcomes. Radiomics can contribute to more precise pre-operative lesion classification, personalized treatment, and cost-effective long-term management. However, there is still a long way to go before radiomic analysis can be routinely used in patient management and clinical decision making. Most of the available data relies on studies with a relatively limited sample size, and the applicability of the technique is essentially limited to macroadenomas. Moreover, a wide variety of software and modes for tumor segmentation and feature extraction exists, and several concerns related to standardization, data heterogeneity, and reproducibility are still present. In the future, new studies with larger patient cohorts are needed as well as a greater harmonization of tumor segmentation and feature extraction protocols, in order to move this technique towards an effective possibility of implementation in clinical practice.

## Figures and Tables

**Table 1:** Summary of the main fields of application of radiomic analyses in PitNETs, with specifically evaluated endpoints and examples of studies included in this review. Abbreviations: CE-T1WI, contrast-enhanced T1-weighted imaging; CS, cavernous sinus; DA, dopamine agonist; DCE-MRI, dynamic contrast-enhanced magnetic resonance imaging; MRI, magnetic resonance imaging; N, number; NCA, null-cell adenoma; NFPA, non-functioning pituitary adenoma; PitNET, pituitary neuroendocrine tumor; RT, radiotherapy; SCA, silent corticotroph adenoma; SRL, somatostatin receptor ligand; SS, sphenoid sinus; T1WI, T1-weighted imaging; T2WI, T2-weighted imaging.

General Field of Application	Specific Endpoint	Studies (First Author, Year)	N. of Patients	PitNET Subtypes	Evaluated MRI Sequences	Software Used for Feature Extraction
Prediction of tumor consistency	Distinction between soft and fibrous tumors	Zeynalova, 2019 [6]	55	Any	T2WI	PyRadiomics
		Cuocolo, 2020 [12]	89	Any	T2WI	PyRadiomics
		Wan, 2022 [11]	156	Any	T1WI, T2WI, CE-T1WI	MatLab
		Fan, 2019 [16]	188	Acromegaly	T1WI, T2WI, CE-T1WI	PyRadiomics
Prediction of tumor invasiveness	Prediction of CS invasion	Niu, 2019 [19]	194	Any	T2WI, CE-T1WI	MatLab
		Zhang, 2022 [20]	196	Any	CE-T1WI	PyRadiomics
	Prediction of CS, SS, or dura mater invasion	Liu, 2020 [21]	50	Any	DCE-MRI	Omni-Kinetics
Prediction of hormonal secretion patterns	Distinction among hormone secretion profiles	Baysal, 2022 [22]	130	Any	T2WI	PyRadiomics
Prediction of histopathological features	Distinction among Tpit, Pit-1, and SF-1 subfamilies	Peng, 2020 [23]	235	Any	T1WI, T2WI, CE-T1WI	PyRadiomics
	Distinction between SCAs and other NFPA subtypes	Rui, 2022 [24]	302	NFPAs	T1WI, T2WI, CE-T1WI	PyRadiomics
		Wang, 2023 [25]	295	NFPAs	T1WI, T2WI, CE-T1WI	PyRadiomics
	Distinction between NCAs and other NFPA subtypes	Zhang, 2018 [26]	112	NFPAs	T1WI, CE-T1WI	MatLab
	Distinction between densely and sparsely granulated adenomas	Park, 2020 [27]	69	Acromegaly	T2WI	PyRadiomics
		Liu, 2021 [28]	49	Acromegaly	T1WI, T2WI, CE-T1WI	PyRadiomics
	Prediction of a high proliferative index (Ki67 ≥ 3%) ^a^	Ugga, 2019 [29]	89	Any	T2WI	PyRadiomics
		Li, 2023 [30]	1214	Any	T1WI, T2WI, CE-T1WI	PyRadiomics
		Wang, 2023 [31]	246	Any	CE-T1WI	LIFEx
		Fan, 2020 [32]	138	Acromegaly	T1WI, T2WI, CE-T1WI	PyRadiomics
Prediction of response to surgical treatment	Prediction of post-surgical recurrence or regrowth	Galm, 2018 [34]	78	NFPAs	T1WI	ImageJ
		Machado, 2020 [35]	27	NFPAs	CE-T1WI	PyRadiomics
		Zhang, 2020 [36]	50	NFPAs	T2WI, CE-T1WI	Python
		Zhang, 2022 [37]	168	Any	CE-T1WI	PyRadiomics (3D-Slicer extension)
	Prediction of post-surgical biochemical remission	Fan, 2019 [38]	163	Functional adenomas	T1WI, T2WI, CE-T1WI	MatLab
Prediction of response to non-surgical therapies	Response to first-generation SRLs	Kocak, 2019 [44]	47	Acromegaly	T2WI	PyRadiomics
		Galm, 2020 [45]	64	Acromegaly	T1WI	ImageJ
	Response to DAs	Park, 2021 [46]	177	Prolactinomas	T2WI	PyRadiomics
	Response to RT	Fan, 2019 [47]	57	Acromegaly	T1WI, T2WI, CE-T1WI	PyRadiomics

^a^ In the study by Wang et al. (2023) [31], the definition of a high proliferation/aggressive behavior was based on the presence of at least two of the following three characteristics: Ki67 ≥ 3%, high mitotic count (≥2/10 high power fields), or positive staining for p53.

## Data Availability

No new data were created or analyzed in this study. Data sharing is not applicable to this article.
